# Hypothermia and recovery outcomes before and after implementation of a multimodal perioperative temperature-management bundle in minimally invasive radical rectal cancer surgery

**DOI:** 10.3389/fonc.2026.1888554

**Published:** 2026-08-27

**Authors:** Qian Liu, Ling Liu, Chenxi Han, Chunmei Cao, Jiefang Ji, Aihua Zhu

**Affiliations:** 1 Department of Anesthesiology and Operating Room, Chengyang District People’s Hospital, Qingdao, Shandong, China; 2 Department of Gastrointestinal Surgery, Huai’an Hospital of Huai’an City (Huai’an Clinical Medical College of Jiangsu University), Huai’an, Jiangsu, China; 3 Department of Operating Room, Nantong Tongzhou Hospital of Traditional Chinese Medicine, Nantong, Jiangsu, China

**Keywords:** hypothermia, minimally invasive surgical procedures, patient care bundles, perioperative care, rectal neoplasms

## Abstract

**Background:**

Inadvertent perioperative hypothermia is common during minimally invasive rectal cancer surgery. We assessed whether a five-component multimodal temperature-management bundle was associated with improved perioperative outcomes.

**Methods:**

This single-center retrospective before-and-after study included 247 adults undergoing laparoscopic or robot-assisted radical rectal cancer surgery from 2021 to 2025 (pre-implementation, n = 123; post-implementation, n = 124). The primary outcome was any intraoperative nasopharyngeal core temperature <36.0 °C. Key secondary outcomes were minimum temperature, cumulative hypothermia duration, postanesthesia care unit (PACU) stay, shivering, and 30-day composite complications. Prespecified mixed-effects and multiplicity-adjusted analyses were performed.

**Results:**

Hypothermia occurred in 84/123 patients (68.3%) before implementation and 19/124 (15.3%) after implementation (absolute risk difference, −53.0 percentage points; 95% confidence interval [CI], −62.2 to −41.6; *P* < 0.001). After implementation, minimum temperature was higher (36.05 ± 0.22 °C vs 35.28 ± 0.62 °C), hypothermia duration was shorter (0 [0–0] vs 42 [0–88] min), PACU stay was shorter (50 [43–60] vs 66 [55–79] min), shivering was less frequent (7.3% vs 42.3%), and composite complications were lower (17.7% vs 36.6%) (all adjusted *P* ≤ 0.001). The implementation period remained associated with lower hypothermia odds after adjustment (adjusted odds ratio, 0.066; 95% CI, 0.029–0.149).

**Conclusions:**

Bundle implementation was associated with lower intraoperative hypothermia burden and improved selected recovery outcomes. The nonrandomized calendar-period design and possible secular confounding limit causal interpretation.

## 1 Introduction

Rectal cancer is a common gastrointestinal malignancy worldwide, and its disease burden remains substantial (1). Laparoscopic and robot-assisted radical rectal cancer surgery can reduce incisional trauma while preserving oncologic principles and has become an important component of minimally invasive surgery (2). Core-to-peripheral heat redistribution after induction of general anesthesia, sustained pneumoperitoneum, prolonged operative duration, abdominal exposure, and fluid administration can jointly increase the risk of inadvertent perioperative hypothermia (3). Minimally invasive surgery does not eliminate this risk; longer anesthesia duration, lower body mass index, and lower baseline temperature remain associated with intraoperative hypothermia (4).

A core temperature below 36.0 °C may be accompanied by shivering, delayed emergence, altered drug metabolism, and coagulation-related physiologic changes; standardized monitoring and active thermal management are therefore important components of perioperative safety (5). Existing evidence indicates that a single warming intervention can improve the temperature trajectory in some patients, but may provide limited coverage of heat loss caused by anesthetic redistribution, cutaneous heat dissipation, infusion of cold fluids, and pneumoperitoneum (6).

Risk of perioperative hypothermia is markedly heterogeneous. Existing models have identified high-risk patients using clinical variables from laparoscopic surgical populations (7), whereas other models have incorporated perioperative data to predict intraoperative hypothermia (8). Prediction studies in gastrointestinal surgery have identified baseline or start-of-surgery temperature, operative duration, and anesthesia duration as variables with high predictive importance (9). A large study of noncardiac surgery further integrated the depth and duration of temperature reduction into a measure of hypothermia burden, suggesting that time-temperature information may complement a single-threshold outcome (10).

Risk prediction and evaluation of an institutional implementation strategy address different clinical questions. The coordinated use of ambient-temperature control, active surface warming, warming of intravenous and irrigation fluids, warmed and humidified carbon dioxide insufflation, and low-flow anesthesia may reduce heat loss through multiple pathways. We therefore conducted a single-center retrospective before-and-after cohort study to compare intraoperative hypothermia and recovery outcomes in patients undergoing minimally invasive radical rectal cancer surgery before and after implementation of a multimodal bundle. The primary outcome was any valid nasopharyngeal core temperature below 36.0 °C during surgery. Temperature trajectories, hypothermia burden, recovery in the postanesthesia care unit (PACU), 30-day complications, and exploratory laboratory outcomes were also assessed.

## 2 Materials and methods

### 2.1 Study design and participants

This was a single-center retrospective before-and-after cohort study reported in accordance with the Strengthening the Reporting of Observational Studies in Epidemiology (STROBE) recommendations (11). We consecutively screened adults who underwent laparoscopic or robot-assisted radical rectal cancer surgery at Nantong Tongzhou Hospital of Traditional Chinese Medicine between January 2021 and December 2025. The study protocol was approved by the institutional ethics committee (approval no. 2025054), which waived the requirement for written informed consent for this retrospective analysis.

Inclusion criteria were as follows: (1) pathologically confirmed rectal adenocarcinoma; (2) age 18–80 years; (3) American Society of Anesthesiologists (ASA) physical status I–III; (4) Dixon or Miles procedure; (5) recorded operative duration ≥ 120 min; and (6) preinduction tympanic temperature of 36.0–37.2 °C. Body mass index (BMI) was calculated as weight (kg)/height squared (m²). Exclusion criteria were active preoperative infection or fever, a known disorder affecting thermoregulation, conversion to open surgery, intraoperative blood loss > 1000 mL or need for emergency hemodynamic intervention, another active malignancy, or > 20% missing key temperature records.

### 2.2 Exposure definition and temperature-management protocols

Patients treated in 2021–2023 were classified as the pre-implementation cohort (*n* = 123), and those treated in 2024–2025 as the post-implementation cohort (*n* = 124), according to the date on which the institutional multimodal temperature-management bundle was introduced. Thus, exposure was assigned by calendar period in a nonconcurrent before-and-after comparison.

During the pre-implementation period, no systematic preventive warming bundle was in place. Operating-room temperature was maintained at 22–24 °C and relative humidity at 50%–60%; intravenous fluids and intraperitoneal irrigation solutions were used at room temperature; and cotton drapes were used routinely, without routine preventive active warming. Forced-air warming was initiated when nasopharyngeal core temperature remained < 36.0 °C for 15 min and was initiated immediately when core temperature fell below 35.5 °C. Patients with a transient temperature below 36.0 °C who did not meet the duration or nadir trigger were closely monitored.

During the post-implementation period, a five-component bundle was applied: (1) operating-room temperature of 24–26 °C until completion of anesthesia induction and 23–25 °C thereafter; (2) forced-air warming at 43 °C from the start of induction together with underbody circulating-water warming at 38–40 °C; (3) warming of intravenous fluids and blood products to 37 °C and prewarming of intraperitoneal irrigation solution to 37–38 °C; (4) warming carbon dioxide (CO_2_) to 37 °C and humidifying it to > 95% relative humidity before insufflation; and (5) use of a low-flow anesthesia circuit with fresh-gas flow ≤ 1 L/min and a heat-and-moisture exchanger. When hypothermia occurred, device output, warming coverage, and tubing connections were checked, and active warming was continued. Skin integrity was assessed before draping, during position changes, and at the end of surgery.

No major planned changes were identified between periods in the principal surgical approaches, anesthetic maintenance techniques, or team composition; nevertheless, comparisons across calendar years may remain susceptible to unmeasured secular factors.

### 2.3 Temperature measurement, data extraction, and quality control

Preinduction tympanic temperature (T_0_) was measured within 30 min before anesthesia induction using the same model of calibrated tympanic thermometer in the same ear canal; invalid device readings or abnormal recordings were remeasured immediately. After tracheal intubation, a nasopharyngeal temperature probe was positioned in the posterior nasopharynx. Once position and signal stability had been confirmed, the anesthesia information management system (AIMS) automatically stored measurements at 1-min intervals until the probe was removed near the end of surgery. Nasopharyngeal temperature reflects core temperature during general anesthesia (12). Prespecified intraoperative time points were 30 min after induction (T_1_), 60 min after skin incision (T_2_), 120 min after skin incision (T_3_), and the end of surgery (T_4_). For each time point, the median of valid observations within ± 2 min of the target time was used.

Quality-control rules excluded values < 33.0 °C or > 40.0 °C, values changing by > 0.3 °C within 1 min when probe displacement was documented, and records flagged by AIMS as probe disconnection. For consecutive gaps ≤ 3 min, linear interpolation was used only to calculate cumulative time with core temperature < 36.0 °C; the primary outcome, minimum intraoperative temperature, and prespecified time-point values were determined from valid observed measurements. An interpolation sensitivity check did not alter primary-outcome classification. Patients with > 20% missing key temperature records were excluded at screening. Non-temperature variables and covariates in the multivariable models were complete in the included cohort; no statistical imputation was performed.

### 2.4 PACU observation, rescue warming, and discharge criteria

T_5_ was defined as tympanic temperature measured within 5 min after admission to the postanesthesia care unit (PACU), and T_6_ as the final tympanic temperature before PACU discharge. Both measurements were obtained in the same ear canal with the same model of calibrated tympanic thermometer; invalid device readings or abnormal records were remeasured immediately. Tympanic temperature was reassessed every 15 min in the PACU, and patients with an admission temperature < 36.0 °C received immediate forced-air warming. Shivering was graded using the Crossley–Mahajan 0–4 scale (13): 0, no shivering; 1, piloerection or peripheral vasoconstriction without visible muscular activity; 2, muscular activity in one muscle group only; 3, muscular activity in more than one muscle group without generalized shivering; and 4, intense generalized shivering. Shivering was defined as a grade ≥ 1. Agitation and sedation were assessed using the Riker Sedation–Agitation Scale (SAS) (14). A score of 4 indicates a calm and cooperative patient; SAS ≥ 5 was defined as agitation and SAS ≤ 3 as a sedated state. PACU discharge required all of the following: a modified Aldrete score ≥ 9 (15), stable circulatory and respiratory status, tympanic temperature ≥ 36.0 °C, shivering grade ≤ 1, and tolerable pain and nausea/vomiting after treatment.

### 2.5 Outcome definitions

The primary outcome was intraoperative hypothermia, defined as any quality-controlled core temperature < 36.0 °C between the first stable nasopharyngeal recording after tracheal intubation and probe removal.

Key secondary outcomes were minimum intraoperative temperature in the full cohort, cumulative time with core temperature < 36.0 °C, PACU length of stay, incidence of shivering, and 30-day composite complications. Other secondary outcomes included the T_1_–T_4_ temperature trajectory, temperature at PACU admission and discharge, PACU agitation, sedated state, rescue analgesia, postoperative nausea and vomiting, recovery milestones, resting Numeric Rating Scale (NRS) pain scores, and oral morphine milligram equivalents (MME). The NRS assesses postoperative pain intensity on a 0–10 scale, with 0 indicating no pain and 10 the worst imaginable pain (16). Hemoglobin (Hb), platelet count (PLT), prothrombin time (PT), activated partial thromboplastin time (APTT), fibrinogen (FIB), C-reactive protein (CRP), interleukin-6 (IL-6), and procalcitonin (PCT) were defined as exploratory laboratory outcomes. Metrics from the full cohort were used to characterize hypothermia burden, whereas a conditional subgroup of patients who developed hypothermia was used to describe event severity.

### 2.6 Postoperative complications and follow-up

Complications were assessed from the end of surgery through postoperative day 30. Incisional surgical-site infection included superficial or deep incisional infection and was adjudicated on the basis of purulent drainage, microbiologic evidence, opening or drainage of the incision, or a clinical diagnosis. Pulmonary infection required a new or progressive radiographic infiltrate plus at least one of fever, abnormal leukocyte count, purulent respiratory secretions, or worsening oxygenation. Urinary tract infection required urinary symptoms or otherwise unexplained fever supported by urine culture (17). Anastomotic leakage was assessed only after Dixon procedures and was defined as a defect in bowel-wall integrity at the anastomosis that created communication between the intra- and extraluminal compartments; clinical, radiologic, endoscopic, or reoperation findings could establish the diagnosis (18). Suspected deep vein thrombosis required confirmation by lower-extremity compression ultrasonography. Cardiovascular events included myocardial infarction, new-onset arrhythmia requiring treatment, acute heart failure, or stroke. Composite complications were counted per patient; multiple events in the same patient contributed one composite event, while individual complications were counted separately. Staging followed the eighth-edition American Joint Committee on Cancer (AJCC) tumor-node-metastasis (TNM) framework, and rectal cancer imaging and terminology were standardized according to the 2023 lexicon (19).

During hospitalization, progress notes, laboratory and imaging results, and records of reintervention were reviewed daily. After discharge, follow-up was conducted in the clinic or by structured telephone interview at postoperative days 7 ± 2 and 30 ± 3. Only events occurring from the end of surgery through postoperative day 30 were included. Two investigators adjudicated outcomes independently, with disagreements resolved by a senior surgeon. Postoperative opioid consumption was converted to oral MME using a standardized conversion method (20).

### 2.7 Statistical analysis

Analyses were performed using IBM SPSS Statistics version 26.0 and R version 4.4.2; all tests were two-sided. Normally distributed continuous variables were presented as mean ± standard deviation (SD) and compared using the independent-samples *t* test; Welch’s *t* test was used when variances were unequal. Clearly skewed continuous variables or variables truncated by a clinical threshold were presented as median and interquartile range (IQR; 25th–75th percentile) and compared using the Mann–Whitney *U* test. Categorical variables were presented as number (percentage) and compared using the Pearson *χ²* test; Fisher’s exact test was used when expected cell counts were small. Baseline balance was also described using the standardized mean difference (SMD), with an absolute value < 0.10 indicating a small difference. Multicategory baseline variables were dummy coded, and the maximum absolute SMD across categories was reported.

For the primary outcome, we reported the incidence, the Newcombe–Wilson absolute risk difference with 95% confidence interval (CI), and the relative risk (RR) with 95% CI, without multiplicity adjustment. Because T_1_–T_4_ were all nasopharyngeal measurements, they were analyzed in a repeated-measures mixed-effects model fitted by restricted maximum likelihood. Fixed effects were group, time, and the group-by-time interaction. Repeated observations within patients were treated as correlated; among unstructured, compound-symmetry, and first-order autoregressive covariance structures, the unstructured form was selected according to the Akaike information criterion. Degrees of freedom were approximated using the Satterthwaite method. Model residuals were assessed using quantile–quantile (Q–Q) and residual-versus-fitted plots. Between-group effects at each time point were reported as model-estimated marginal mean differences with 95% CIs. The Holm method controlled the family-wise error rate across the five key secondary outcomes. Other PACU outcomes, recovery outcomes, pain outcomes, individual complications, and 16 postoperative laboratory comparisons were adjusted within clinically defined outcome families using the Benjamini–Hochberg false discovery rate (FDR), with FDR-adjusted *P* values reported as *q* values. The three comparisons in the conditional hypothermia subgroup and the two supplementary ordinal-scale comparisons were each adjusted using the Holm method. All multiplicity adjustments used unrounded raw *P* values.

No variables were selected according to univariable *P* values, and no stepwise procedure was used. The extended clinical association model prespecified implementation period, age, sex, BMI, ASA physical status III, T_0_ temperature, operative duration, and total intraoperative intravenous fluid volume; all eight variables were entered simultaneously into a binary logistic regression model. Effects are reported as odds ratios (ORs) with 95% CIs. The model estimates the period association conditional on operative duration and fluid volume. Age was scaled per 10 years, T_0_ per 0.1 °C, operative duration per 30 min, and fluid volume per 200 mL. Because operative duration and fluid volume were measured after implementation-period assignment, a baseline-only sensitivity model was also fitted using implementation period, age, sex, BMI, ASA physical status III, T_0_ temperature, Dixon versus Miles procedure, and laparoscopic versus robotic approach. The linearity of relationships between continuous variables and the logit was examined, as were variance inflation factors. Robustness was assessed using Firth-penalized logistic regression and 1000 bootstrap coefficient resamples.

## 3 Results

### 3.1 Participant flow, baseline characteristics, and perioperative characteristics

**Figure 1:**
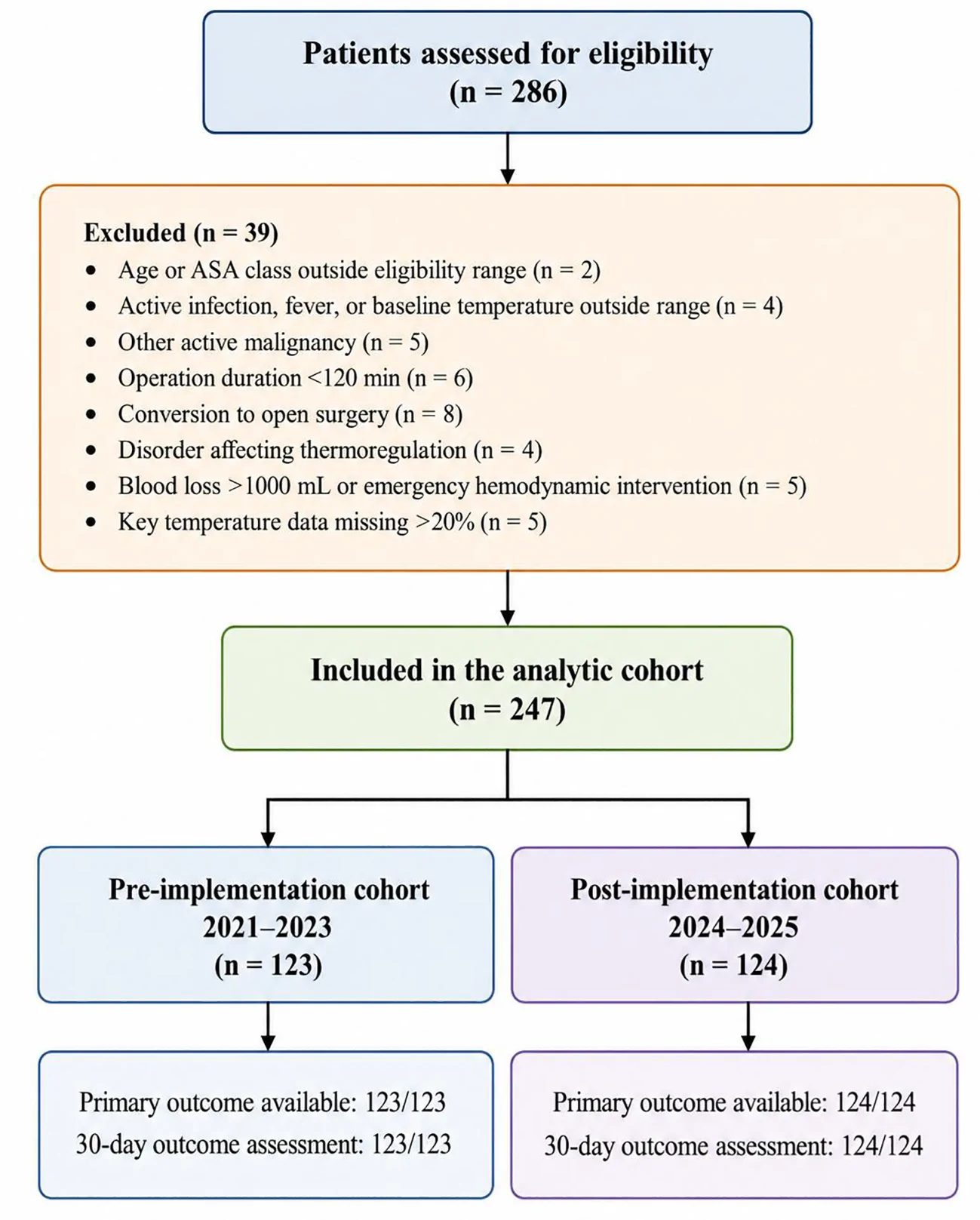
Flow diagram of patient screening and analysis. Patients who underwent minimally invasive radical rectal cancer surgery between January 2021 and December 2025 were screened consecutively. Exclusion reasons were counted as mutually exclusive and totaled 39; 123 patients in the pre-implementation cohort and 124 in the post-implementation cohort entered the primary analysis and the 30-day outcome analysis. n, number of patients; ASA, American Society of Anesthesiologists physical status; AIMS, anesthesia information management system.

Of 286 patients screened, 39 were excluded for mutually exclusive reasons: age or ASA physical status outside the eligibility range (*n* = 2); active infection or fever, or baseline temperature outside the eligible range (*n* = 4); another active malignancy (*n* = 5); operative duration < 120 min (*n* = 6); conversion to open surgery (*n* = 8); a disorder affecting thermoregulation (*n* = 4); blood loss > 1000 mL or emergency hemodynamic intervention (*n* = 5); and > 20% missing key temperature records (*n* = 5). The final cohort comprised 247 patients, including 123 in the pre-implementation cohort and 124 in the post-implementation cohort. Thirty-day outcome assessment was complete in both groups (100% in each; **Figure 1**).

Between-group differences were small for the proportion of men, age, height, weight, BMI, ASA physical status, hypertension, diabetes mellitus, coronary artery disease, chronic obstructive pulmonary disease, TNM stage, tumor distance from the anal verge, Dixon versus Miles procedure, laparoscopic versus robotic approach, and T_0_ tympanic temperature (all *P* > 0.05; the maximum category-specific absolute SMD for multicategory variables and all other absolute SMDs were < 0.10). Operative duration, anesthesia duration, pneumoperitoneum duration and pressure, total CO_2_ use, anesthetic maintenance technique, induction drugs, total intravenous fluid volume, crystalloid volume, colloid volume, proportion receiving intraoperative red blood cell transfusion, intraoperative blood loss, urine output, mean arterial pressure, and heart rate did not differ significantly between groups (all *P* > 0.05). Transfusion volume among the small number of patients who received red blood cells is reported descriptively (**Table 1**).

**Table 1:** Baseline and perioperative characteristics.

Variable	Pre-implementation cohort (*n* = 123)	Post-implementation cohort (*n* = 124)	Test statistic	*P* value	Absolute SMD (maximum category-specific SMD for multicategory variables)
Male sex, *n* (%)	74 (60.2)	75 (60.5)	*χ²* = 0.003	0.959	0.007
Age (years)	62.3 ± 9.8	61.8 ± 10.2	*t* = 0.393	0.695	0.050
Height (cm)	165.4 ± 7.6	166.1 ± 7.3	*t* = −0.738	0.461	0.094
Weight (kg)	63.8 ± 10.2	64.5 ± 10.8	*t* = −0.524	0.601	0.067
BMI (kg/m²)	23.3 ± 2.9	23.4 ± 3.1	*t* = −0.262	0.794	0.033
ASA physical status I/II/III, *n*	18/81/24	17/83/24	*χ²* = 0.049	0.976	0.027
Hypertension, *n* (%)	48 (39.0)	47 (37.9)	*χ²* = 0.033	0.856	0.023
Diabetes mellitus, *n* (%)	28 (22.8)	27 (21.8)	*χ²* = 0.035	0.852	0.024
Coronary artery disease, *n* (%)	14 (11.4)	13 (10.5)	*χ²* = 0.051	0.821	0.030
Chronic obstructive pulmonary disease, *n* (%)	9 (7.3)	8 (6.5)	*χ²* = 0.072	0.788	0.034
TNM stage I/II/III, *n*	31/52/40	32/51/41	*χ²* = 0.034	0.983	0.023
Tumor distance from the anal verge (cm)	7.8 ± 3.2	7.6 ± 3.4	*t* = 0.476	0.634	0.061
Dixon/Miles procedure, *n*	98/25	99/25	*χ²* = 0.001	0.974	0.004
Laparoscopic/robotic approach, *n*	102/21	103/21	*χ²* = 0.001	0.977	0.004
T_0_ tympanic temperature (°C)	36.52 ± 0.21	36.54 ± 0.22	*t* = −0.731	0.466	0.093
Operative duration (min)	187.6 ± 34.2	184.3 ± 33.8	*t* = 0.763	0.446	0.097
Anesthesia duration (min)	218.4 ± 38.5	215.7 ± 37.2	*t* = 0.560	0.576	0.071
Pneumoperitoneum duration (min)	158.3 ± 31.6	155.8 ± 30.9	*t* = 0.629	0.530	0.080
Pneumoperitoneum pressure (mmHg)	12.8 ± 1.1	12.7 ± 1.0	*t* = 0.747	0.456	0.095
Total CO_2_ use (L)	132.4 ± 28.7	130.2 ± 27.9	*t* = 0.611	0.542	0.078
Total intravenous anesthesia/combined intravenous–inhalational anesthesia, n	38/85	39/85	*χ²* = 0.009	0.925	0.012
Propofol/etomidate/other induction agent, n	86/29/8	87/28/9	*χ²* = 0.078	0.962	0.030
Total intravenous fluid volume (mL)	1823.6 ± 387.4	1798.4 ± 372.6	*t* = 0.521	0.603	0.066
Crystalloid volume (mL)	1398.5 ± 312.3	1381.7 ± 308.4	*t* = 0.425	0.671	0.054
Colloid volume (mL)	425.1 ± 142.6	416.7 ± 138.9	*t* = 0.469	0.640	0.060
Intraoperative red blood cell transfusion, *n* (%)	6 (4.9)	5 (4.0)	Fisher’s exact test	0.769	0.041
Red blood cell transfusion volume (mL; transfused patients only)	300 (300–450)	300 (300–450)	Descriptive	—	—
Intraoperative blood loss (mL)	142.3 ± 68.5	138.7 ± 65.2	*t* = 0.423	0.673	0.054
Urine output (mL)	387.4 ± 142.6	394.2 ± 148.3	*t* = −0.367	0.714	0.047
Mean arterial pressure (mmHg)	82.4 ± 11.3	83.1 ± 10.8	*t* = −0.498	0.619	0.063
Heart rate (beats/min)	74.2 ± 10.6	73.8 ± 11.2	*t* = 0.288	0.773	0.037

Data are presented as mean ± standard deviation (SD), median (interquartile range [IQR]), or number (percentage). Continuous variables were compared using the independent-samples *t* test according to distribution; categorical variables were compared using the Pearson *χ²* test, or Fisher’s exact test when expected cell counts were small. The standardized mean difference (SMD) describes baseline balance; multicategory variables were dummy coded, and the maximum absolute SMD across categories is reported. BMI, body mass index; ASA, American Society of Anesthesiologists physical status; TNM, tumor-node-metastasis; T_0_, tympanic temperature before anesthesia induction; CO_2_, carbon dioxide; *n*, number of patients. Fresh-gas flow and warming adherence are reported in **Supplementary Table 1**. Red blood cell transfusion volume is limited to patients who actually received transfusion and is reported descriptively because of the small sample.

### 3.2 Primary outcome and intraoperative temperature burden

The AIMS 1-min temperature series had a high proportion of valid records and a low proportion of missing or artifactual values. Intermittent gaps were within the prespecified short-gap limit, all T_1_–T_4_ windows were complete, patients with a high proportion of missing key temperature data had been excluded at screening, and interpolation of short gaps did not change primary-outcome classification. Residual diagnostics for the repeated-measures mixed model showed no substantial deviation. Most hypothermic patients in the pre-implementation cohort received rescue warming after meeting a trigger; patients who did not receive rescue warming met neither the duration nor the nadir trigger. The interval from first hypothermia to warming initiation included the trigger-observation period, whereas device deployment after trigger fulfillment was brief; rescue-warming duration is described only among treated patients. All hypothermic patients in the post-implementation cohort underwent checks of device function and warming coverage. In the post-implementation cohort, forced-air and circulating-water warming covered most of the perioperative period, average fluid-warmer and CO_2_-device output temperatures approximated their targets, mean CO_2_ humidity exceeded the target of 95%, and fresh-gas flow was lower than in the pre-implementation cohort (*P* < 0.001). In the pre-implementation cohort, hypothermia commonly persisted to PACU admission, and new hypothermia also occurred during transfer; neither pattern was observed after implementation (**Supplementary Table 1**).

**Figure 2:**
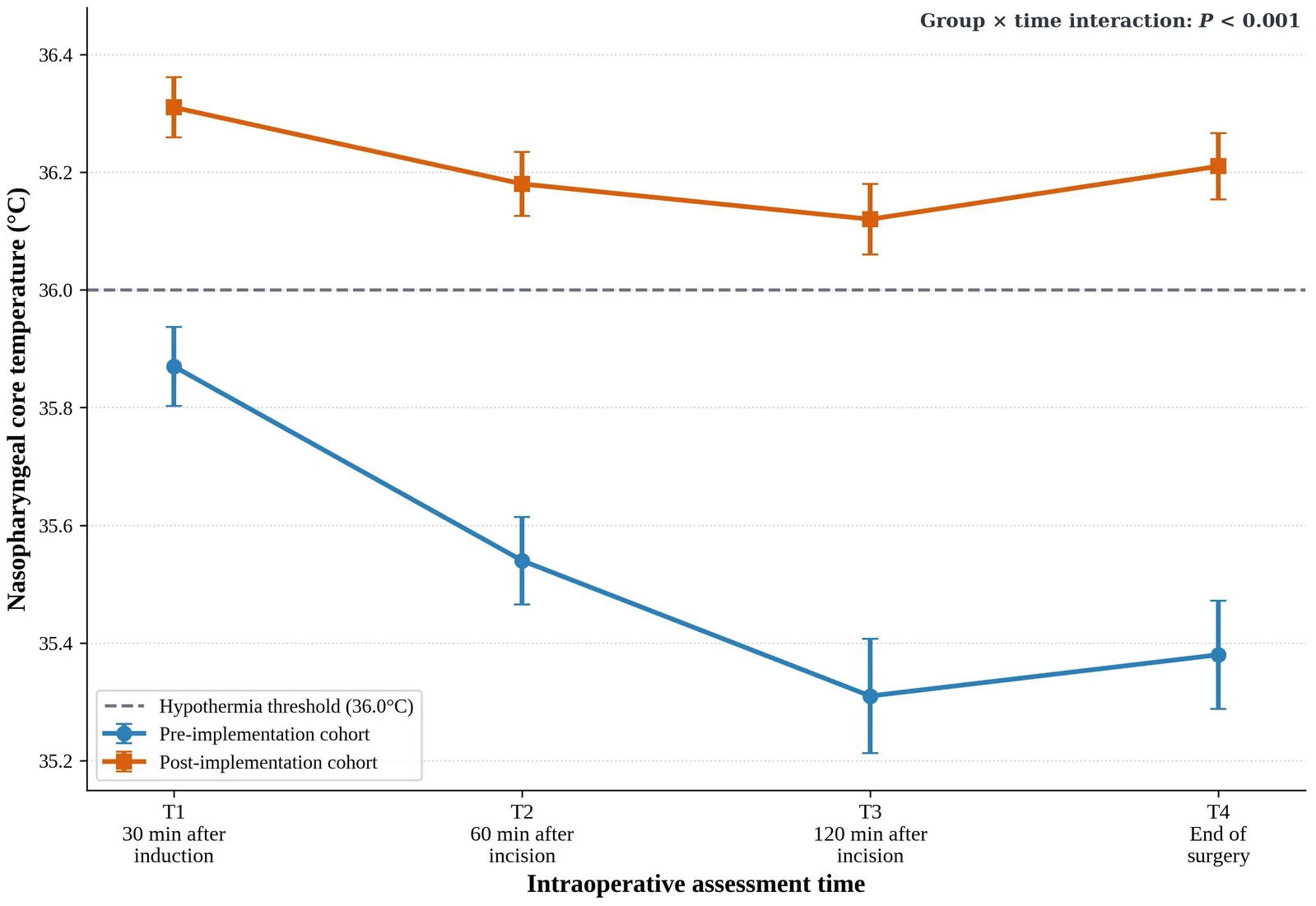
Intraoperative nasopharyngeal core temperature trajectories in the pre-implementation and post-implementation cohorts. Points are model-estimated marginal means from the repeated-measures mixed-effects model; error bars are 95% confidence intervals (CIs), and the dashed line denotes the 36.0 °C hypothermia threshold. T_1_, T_2_, T_3_, and T_4_ correspond to 30 min after anesthesia induction, 60 min after skin incision, 120 min after skin incision, and the end of surgery, respectively. The model included fixed effects for group, time, and group-by-time interaction, with an unstructured covariance matrix for within-patient correlation; *post hoc* between-group comparisons at T_1_–T_4_ were adjusted using the Holm method. T_0_ was the preinduction tympanic temperature; T_5_ was the tympanic temperature within 5 min after admission to the postanesthesia care unit (PACU); and T_6_ was the final tympanic temperature before PACU discharge. T_0_, T_5_, and T_6_ were not included in this trajectory.

The incidence of intraoperative hypothermia was lower after implementation; both the absolute risk difference and relative risk indicated a marked reduction (*P* < 0.001). The time effect, group effect, and group-by-time interaction in the repeated-measures mixed model were all significant (all *P* < 0.001). Nasopharyngeal core temperatures at T_1_, T_2_, T_3_, and T_4_ were higher in the post-implementation cohort (all Holm-adjusted *P* < 0.001). Across all patients, minimum intraoperative temperature was higher and cumulative time with core temperature < 36.0 °C was shorter after implementation (both Holm-adjusted *P* < 0.001; **Table 2**, **Figure 2**).

**Table 2:** Primary outcome and intraoperative temperature burden.

Outcome	Pre-implementation cohort	Post-implementation cohort/effect estimate	Statistical method/effect	Raw *P*	Adjusted *P*
Intraoperative hypothermia, *n* (%)	84 (68.3)	19 (15.3)	*χ²* = 71.267	< 0.001	Primary outcome; no adjustment
Absolute risk difference (post − pre), percentage points	—	−53.0 (95% CI, −62.2 to −41.6)	Newcombe–Wilson	< 0.001	Primary outcome; no adjustment
Relative risk	—	0.224 (95% CI, 0.146–0.345)	Log method, 95% CI	< 0.001	Primary outcome; no adjustment
T_1_ nasopharyngeal temperature (°C)	35.87 ± 0.38	36.31 ± 0.29	Model-estimated marginal mean difference, 0.44 (0.36–0.52)	< 0.001	Holm < 0.001
T_2_ nasopharyngeal temperature (°C)	35.54 ± 0.42	36.18 ± 0.31	Model-estimated marginal mean difference, 0.64 (0.55–0.73)	< 0.001	Holm < 0.001
T_3_ nasopharyngeal temperature (°C)	35.31 ± 0.55	36.12 ± 0.34	Model-estimated marginal mean difference, 0.81 (0.70–0.92)	< 0.001	Holm < 0.001
T_4_ nasopharyngeal temperature (°C)	35.38 ± 0.52	36.21 ± 0.32	Model-estimated marginal mean difference, 0.83 (0.72–0.94)	< 0.001	Holm < 0.001
Minimum intraoperative temperature (full cohort, °C)	35.28 ± 0.62	36.05 ± 0.22	Welch’s *t* = −12.987	< 0.001	Holm < 0.001
Cumulative time with temperature < 36.0 °C (full cohort, min)	42 (0–88)	0 (0–0)	Mann–Whitney *U*	< 0.001	Holm < 0.001
Group-by-time interaction	—	—	Repeated-measures mixed-effects model	< 0.001	Global test

Data are presented as mean ± standard deviation (SD), median (interquartile range [IQR]), or number (percentage). CI, confidence interval; RR, relative risk; *n*, number of patients. T_1_, T_2_, T_3_, and T_4_ denote nasopharyngeal core temperature 30 min after anesthesia induction, 60 min after skin incision, 120 min after skin incision, and at the end of surgery, respectively. Intraoperative hypothermia was compared using the Pearson *χ²* test; the absolute risk difference used the Newcombe–Wilson method; the 95% CI for RR used the log method; between-group effects at T_1_–T_4_ are model-estimated marginal mean differences from the repeated-measures mixed-effects model; minimum intraoperative temperature used Welch’s *t* test; and cumulative hypothermia duration used the Mann–Whitney *U* test. The primary outcome was not multiplicity adjusted; *post hoc* comparisons at the four time points and the five key secondary outcomes were adjusted using the Holm method.

Within the conditional subgroup of patients who developed hypothermia, the post-implementation cohort had shorter hypothermia duration and a higher minimum temperature (both Holm-adjusted *P* < 0.001). The distribution of the phase in which the minimum temperature occurred also differed between groups (Fisher–Freeman–Halton exact test, Holm-adjusted *P* = 0.023; **Supplementary Table 2**).

### 3.3 Postanesthesia care unit outcomes

At PACU admission, the post-implementation cohort had a higher T_5_ tympanic temperature and lower proportions of admission hypothermia and rescue forced-air warming. T_6_ discharge temperature was also higher, and no patient in either cohort was discharged with a temperature < 36.0 °C. PACU length of stay was shorter and shivering less frequent after implementation (both Holm-adjusted *P* < 0.001). Agitation and use of rescue analgesics were less frequent after implementation (both *q* < 0.001), whereas sedated state and postoperative nausea and vomiting did not differ significantly (both *q* > 0.05; **Table 3**). The full shivering-grade distribution shifted toward lower grades, and the full SAS distribution was more concentrated at a score of 4; both ordinal-distribution comparisons remained significant after Holm adjustment (both *P* < 0.001). The threshold outcome SAS ≤ 3 did not differ significantly (**Supplementary Table 3**).

**Table 3:** Postanesthesia care unit outcomes.

Outcome	Pre-implementation cohort	Post-implementation cohort	Statistical method	Raw *P*	Adjusted *P*/*q*
T_5_ tympanic temperature within 5 min after PACU admission (°C)	35.72 ± 0.36	36.48 ± 0.27	Welch *t*	< 0.001	BH *q* < 0.001
Hypothermia at PACU admission (< 36.0 °C), *n* (%)	96 (78.0)	5 (4.0)	*χ²* = 139.961	< 0.001	BH *q* < 0.001
Rescue forced-air warming in the PACU, *n* (%)	96 (78.0)	5 (4.0)	Applied according to threshold	—	Descriptive
T_6_ tympanic temperature before PACU discharge (°C)	36.3 (36.2–36.5)	36.5 (36.4–36.7)	Mann–Whitney *U*	< 0.001	BH *q* < 0.001
Temperature < 36.0 °C at PACU discharge, *n* (%)	0	0	Descriptive	—	—
PACU length of stay (min)	66 (55–79)	50 (43–60)	Mann–Whitney *U*	< 0.001	Holm < 0.001
Shivering (Crossley–Mahajan grade ≥ 1), *n* (%)	52 (42.3)	9 (7.3)	*χ²* = 40.717	< 0.001	Holm < 0.001
Agitation (SAS ≥ 5), *n* (%)	40 (32.5)	10 (8.1)	*χ²* = 22.874	< 0.001	BH *q* < 0.001
Sedated state (SAS ≤ 3), *n* (%)	11 (8.9)	20 (16.1)	*χ²* = 2.905	0.088	BH *q* = 0.096
Rescue analgesic use, *n* (%)	38 (30.9)	14 (11.3)	*χ²* = 14.278	< 0.001	BH *q* < 0.001
Postoperative nausea and vomiting, *n* (%)	28 (22.8)	18 (14.5)	*χ²* = 2.772	0.096	BH *q* = 0.096

Data are presented as mean ± standard deviation (SD), median (interquartile range [IQR]), or number (percentage). PACU, postanesthesia care unit; T_5_, tympanic temperature within 5 min after PACU admission; T_6_, final tympanic temperature before PACU discharge; SAS, Riker Sedation–Agitation Scale; BH, Benjamini–Hochberg; FDR, false discovery rate; *q*, FDR-adjusted *P* value; *n*, number of patients. T_5_ was compared using Welch’s *t* test; T_6_ and PACU length of stay were compared using the Mann–Whitney *U* test; categorical outcomes were compared using the Pearson *χ²* test. Rescue warming in the PACU was triggered by T_5_ < 36.0 °C and is reported descriptively. PACU length of stay and shivering were key secondary outcomes and were adjusted using the Holm method; other inferential PACU outcomes were adjusted using the BH FDR procedure.

### 3.4 Thirty-day postoperative complications

Composite complications were less frequent in the post-implementation cohort; the between-group difference remained statistically significant after Holm adjustment, as prespecified for this key secondary outcome. Incisional surgical-site infection and pulmonary infection were numerically less frequent, but the between-group differences did not reach statistical significance after FDR adjustment. Anastomotic leakage, urinary tract infection, deep vein thrombosis, and cardiovascular events did not differ significantly (all *q* > 0.05; **Table 4**).

**Table 4:** Complications within 30 days after surgery.

Complication	Pre-implementation cohort	Post-implementation cohort	Test statistic	Raw *P*	Adjusted *P*/*q*
Incisional surgical-site infection	14/123 (11.4)	5/124 (4.0)	*χ²* = 4.698	0.030	BH *q* = 0.181
Anastomotic leakage†	7/98 (7.1)	5/99 (5.1)	Fisher’s exact test	0.568	BH *q* = 0.568
Pulmonary infection	12/123 (9.8)	5/124 (4.0)	*χ²* = 3.157	0.076	BH *q* = 0.227
Urinary tract infection	8/123 (6.5)	5/124 (4.0)	*χ²* = 0.757	0.384	BH *q* = 0.568
Deep vein thrombosis	5/123 (4.1)	3/124 (2.4)	Fisher’s exact test	0.500	BH *q* = 0.568
Cardiovascular event	4/123 (3.3)	2/124 (1.6)	Fisher’s exact test	0.446	BH *q* = 0.568
Composite complications‡	45/123 (36.6)	22/124 (17.7)	*χ²* = 11.092	< 0.001	Holm-adjusted *P* = 0.001

Data are presented as number (percentage). Categorical outcomes were compared using the Pearson *χ²* test; Fisher’s exact test was used when expected cell counts were small. BH, Benjamini–Hochberg; FDR, false discovery rate; *q*, FDR-adjusted *P* value. †Anastomotic leakage was evaluated only among patients who underwent a Dixon procedure (98 before implementation and 99 after implementation). ‡Composite complications were counted per patient; multiple events in the same patient counted once. Composite complications were a key secondary outcome and were adjusted using the Holm method. The six individual complications constituted a separate BH FDR family.

### 3.5 Recovery, pain, and exploratory laboratory outcomes

Time to first flatus, first oral intake, and first ambulation; urinary catheter duration; time to removal of the intra-abdominal drain; and postoperative length of stay were all shorter after implementation. Resting NRS scores at 24 and 48 h and total postoperative MME were also lower (all *q* < 0.001; **Table 5**).

**Table 5:** Recovery and pain outcomes.

Outcome	Pre-implementation cohort	Post-implementation cohort	Statistical method	Raw *P*	Adjusted *q*
Time to first flatus (h)	70 (60–84)	58 (48–70)	Mann–Whitney *U*	< 0.001	< 0.001
Time to first oral intake (h)	82 (70–96)	68 (58–80)	Mann–Whitney *U*	< 0.001	< 0.001
Time to first ambulation (h)	22 (18–27)	18 (14–22)	Mann–Whitney *U*	< 0.001	< 0.001
Urinary catheter duration (d)	5 (4–6)	4 (3–5)	Mann–Whitney *U*	< 0.001	< 0.001
Time to intra-abdominal drain removal (d)	6 (5–7)	5 (4–6)	Mann–Whitney *U*	< 0.001	< 0.001
Postoperative length of stay (d)	10 (8–12)	8 (7–9)	Mann–Whitney *U*	< 0.001	< 0.001
Resting NRS at 24 h	4 (3–5)	3 (2–4)	Mann–Whitney *U*	< 0.001	< 0.001
Resting NRS at 48 h	3 (2–4)	2 (2–3)	Mann–Whitney *U*	< 0.001	< 0.001
Total postoperative opioid consumption (mg MME)	40 (30–52)	30 (22–40)	Mann–Whitney *U*	< 0.001	< 0.001

Data are presented as median (interquartile range [IQR]) and were compared using the Mann–Whitney *U* test. Recovery and pain outcomes constituted separate Benjamini–Hochberg false discovery rate (FDR) families; *q* denotes the FDR-adjusted *P* value. NRS, Numeric Rating Scale; MME, oral morphine milligram equivalents; h, hours; d, days.

Preoperative Hb, PLT, PT, APTT, FIB, CRP, IL-6, and PCT did not differ between groups (all *P* > 0.05). At 24 and 72 h after surgery, Hb and FIB were higher, PT and APTT were shorter, and CRP, IL-6, and PCT were lower in the post-implementation cohort. With the exception of PLT, these differences remained significant after FDR adjustment (*q* < 0.05; **Supplementary Table 4**). Intraoperative blood loss, the proportion receiving intraoperative red blood cell transfusion, postoperative 0–24 h intravenous fluid volume, urine output, intra-abdominal drainage volume, recorded 0–24 h fluid balance, and the proportion receiving additional red blood cell transfusion within 0–72 h did not differ significantly (all *P* > 0.05). Because few patients were transfused intraoperatively, transfusion volume is presented descriptively (**Supplementary Table 5**). The multiplicity-control inventory covered the primary outcome; five key secondary outcomes; four intraoperative time points; other PACU outcomes; the conditional subgroup; ordinal scales; individual complications; recovery, pain, and 16 laboratory comparisons. Interpretations in the main text were consistent with the adjusted results (**Supplementary Table 6**).

### 3.6 Factors associated with intraoperative hypothermia

**Figure 3:**
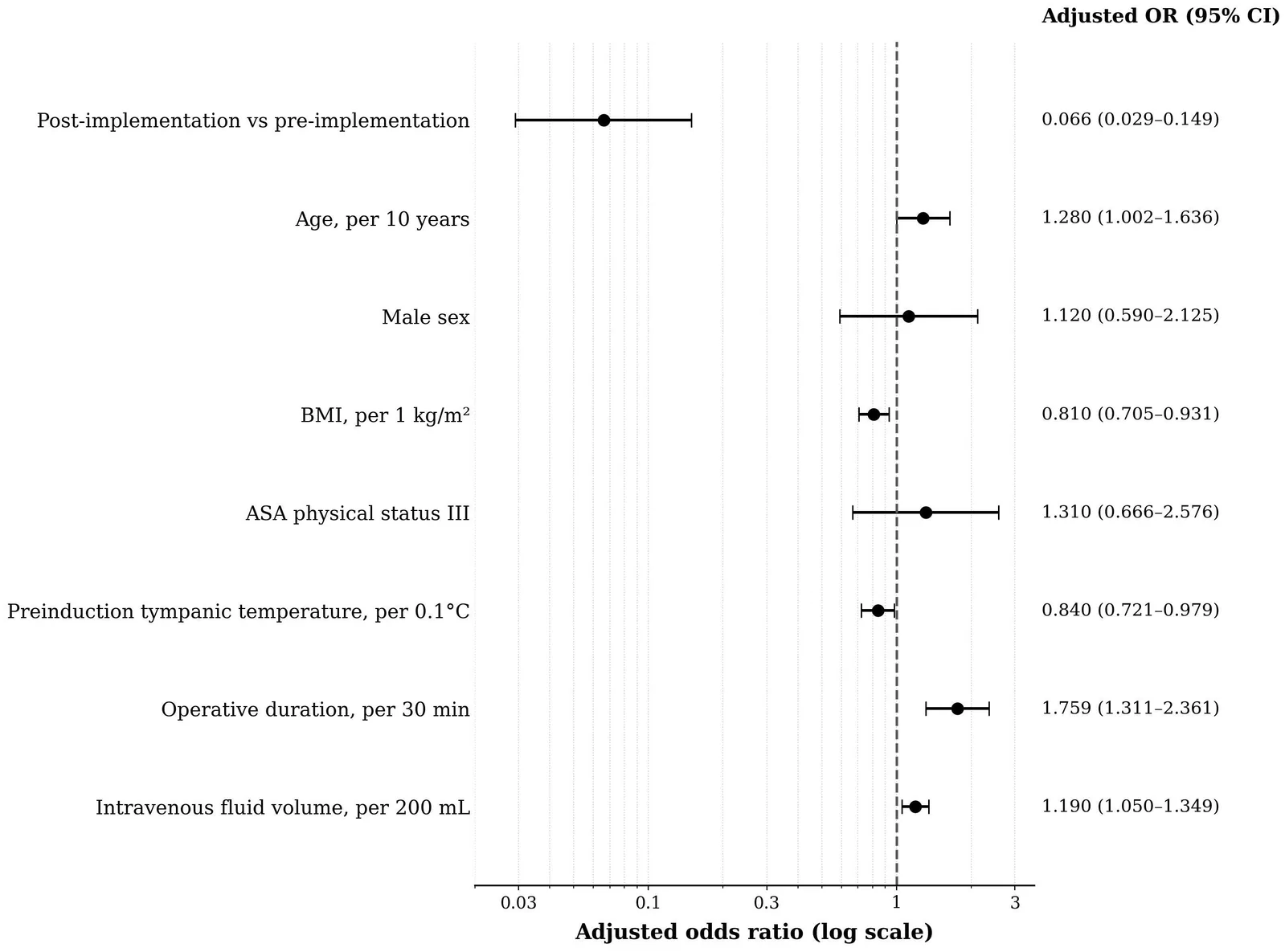
Adjusted odds ratios for factors associated with intraoperative hypothermia. Circles represent adjusted odds ratios (ORs) from the extended clinical association model, and horizontal lines represent 95% confidence intervals (CIs); the dashed line indicates OR = 1. Body mass index (BMI) was scaled per 1 kg/m², age per 10 years, preinduction tympanic temperature (T_0_) per 0.1 °C, operative duration per 30 min, and intravenous fluid volume per 200 mL; American Society of Anesthesiologists (ASA) physical status III was compared with I–II. All variables were entered simultaneously into the model.

The extended clinical association model included 103 hypothermia events and eight predictor parameters, yielding 12.9 events per predictor parameter; no substantial collinearity was detected. Conditional on the other model variables, the post-implementation period was associated with lower odds of intraoperative hypothermia. Older age, longer operative duration, and greater intravenous fluid volume were associated with higher odds, whereas higher BMI and T_0_ temperature were associated with lower odds; sex and ASA physical status III were not statistically significant (**Table 6**, **Figure 3**). The baseline-only sensitivity model, Firth regression, and bootstrap resampling all produced period-effect estimates in the same direction. Diagnostics for linearity of continuous variables and collinearity showed no substantial violation of model assumptions (**Supplementary Table 7**).

**Table 6:** Extended clinical association logistic regression model.

Variable	*β*	SE	Wald *χ²*	Adjusted OR	95% CI	*P* value
Post-implementation vs pre-implementation	−2.718	0.414	43.10	0.066	0.029–0.149	< 0.001
Age (per 10 years)	0.247	0.125	3.90	1.280	1.002–1.636	0.048
Male vs female sex	0.113	0.327	0.12	1.120	0.590–2.125	0.730
BMI (per 1 kg/m²)	−0.211	0.071	8.83	0.810	0.705–0.931	0.003
ASA physical status III vs I–II	0.270	0.345	0.61	1.310	0.666–2.576	0.434
T_0_ (per 0.1 °C)	−0.174	0.078	4.98	0.840	0.721–0.979	0.026
Operative duration (per 30 min)	0.565	0.150	14.19	1.759	1.311–2.361	< 0.001
Intravenous fluid volume (per 200 mL)	0.174	0.064	7.39	1.190	1.050–1.349	0.007

*β*, regression coefficient; SE, standard error; Wald *χ²*, Wald test statistic; OR, odds ratio; CI, confidence interval; BMI, body mass index; ASA, American Society of Anesthesiologists physical status; T_0_, tympanic temperature before anesthesia induction. The dependent variable was intraoperative hypothermia (yes = 1, no = 0), and all variables were entered simultaneously into the binary logistic regression model. Age, T_0_, operative duration, and intravenous fluid volume were scaled per 10 years, 0.1 °C, 30 min, and 200 mL, respectively. Operative duration and fluid volume were measured after implementation-period assignment; therefore, the period OR represents a conditional association.

## 4 Discussion

In this study, the institutional transition from a practice without systematic preventive active warming to a five-component multimodal bundle was associated with a lower incidence and a lower time-temperature burden of intraoperative hypothermia, as well as less need for rescue rewarming in the PACU. During the pre-implementation period, rescue warming was generally initiated only after a threshold was reached, and hypothermia frequently persisted to PACU admission; after implementation, management was preventive and continuous. The extended clinical association model, baseline-only sensitivity model, Firth regression, and bootstrap analysis yielded concordant directions of association, supporting an association between the implementation period and lower hypothermia burden.

Because preventive active warming was not routinely used during the pre-implementation period, the comparison reflects a before-and-after difference between practice without a systematic preventive warming protocol and an intensive multimodal bundle. Recent evidence on optimization of laparoscopic pathways emphasizes that added techniques or processes should be selected according to identifiable clinical benefit (21). Regional analgesic techniques during laparoscopic surgery can also alter pain and recovery (22); consequently, NRS scores, opioid use, and recovery milestones may have been affected concurrently by analgesic pathways, implementation of enhanced recovery after surgery (ERAS), and other secular cointerventions.

The bundle addressed the operating-room environment, body surface, fluids, pneumoperitoneum, and anesthesia circuit, thereby potentially mitigating heat loss from anesthetic redistribution, radiation and convection, infusion of cold fluids, and pneumoperitoneum-related evaporation. Recent pooled evidence indicates that prewarming combined with intraoperative warming is more effective than intraoperative warming alone for maintaining perioperative temperature (23). Warming during induction and administration of warmed intravenous fluids may also attenuate early temperature decline (24). Comparisons of active warming strategies further support continuous thermal management that spans multiple phases in patients at high risk (25).

The directions of association for age, BMI, operative duration, and fluid volume were broadly consistent with evidence on hypothermia risk during laparoscopic surgery (26). Intraoperative hypothermia has also been associated with prolonged PACU stay and poorer quality of recovery (27). The extended model estimated the period association conditional on operative duration and fluid volume, and the baseline-only sensitivity model yielded a consistent direction. These models were used for association estimation; their predictive performance has not been independently validated externally.

The post-implementation cohort had less shivering, a shorter PACU length of stay, and shorter times to recovery milestones, consistent with the coordinated goals of temperature maintenance, early oral intake, early ambulation, and standardized analgesia within colorectal ERAS pathways (28). Reliable implementation of thermal-management protocols depends on equipment availability, staff training, process adherence, and continuous audit (29). The effect of warmed, humidified CO_2_ on temperature maintenance may depend on concomitant surface warming, duration of pneumoperitoneum, and type of surgery (30). The necessary components and cost-effectiveness of the five-component bundle require evaluation in staged or factorial designs.

The between-group difference in composite complications, a key secondary outcome, remained statistically significant after Holm adjustment, whereas no individual complication reached statistical significance after FDR adjustment. Recent pooled evidence has not demonstrated a definitive overall association between intraoperative temperature and surgical-site infection, although risk signals appear stronger at temperatures ≤ 35 °C and in certain surgical subgroups (31). FIB participates in coagulation and also has inflammation-related biologic functions (32). Postoperative Hb is influenced by volume status, transfusion, marrow response, and phlebotomy (33), while visual estimation of blood loss is itself subject to measurement error (34). The recorded 0–24 h fluid balance in this study included only intravenous input, urine output, and intra-abdominal drainage; between-group differences in PT, APTT, FIB, and Hb should therefore be regarded as exploratory laboratory findings.

Evidence on perioperative thermal management in cancer surgery suggests that temperature stability may be associated with recovery and infection-related outcomes, but effects vary according to tumor type, surgical pathway, and cointerventions (35). Continuous active warming during laparoscopic gastric surgery has likewise shown improved temperature control and selected recovery outcomes (36). Multicenter prospective and component-specific studies are still needed in minimally invasive rectal cancer surgery to assess external applicability, implementation costs, and the simplest effective bundle.

### 4.1 Study limitations

This study has several limitations. First, it was a single-center, nonrandomized study with grouping by calendar period; staff experience, ERAS practice, analgesia, infection prevention, and other unmeasured cointerventions may have produced secular confounding. Second, all five components were implemented simultaneously, precluding separate estimation of individual effects and interactions. Third, eligibility required an actual operative duration ≥ 120 min, and patients with intraoperative blood loss > 1000 mL or emergency hemodynamic intervention were excluded. These criteria depend on information available intraoperatively or postoperatively, may introduce selection bias, and limit applicability to shorter procedures and patients with major bleeding or hemodynamic instability. Fourth, five patients were excluded at screening because > 20% of key temperature records were missing; complete-case analysis may still have introduced selection bias. Fifth, tympanic temperature was used preoperatively and in the PACU, whereas nasopharyngeal temperature was used intraoperatively. Measurements from different sites were analyzed separately, but cross-site changes remain susceptible to measurement differences. Sixth, the study relied on retrospective AIMS and medical-record data; quality control can reduce but not eliminate recording error. Seventh, the extended model included operative duration and fluid volume, which were measured after implementation-period assignment, so the period coefficient represents a conditional association. The baseline-only sensitivity model was directionally consistent, but residual confounding may remain. Eighth, the outcome hierarchy was defined according to study objectives and clinical importance but was not prospectively registered; secondary and exploratory outcomes should be interpreted cautiously. Ninth, statistical power was limited for uncommon outcomes such as anastomotic leakage, deep vein thrombosis, and cardiovascular events.

## 5 Conclusions

In this single-center retrospective before-and-after cohort, implementation of a multimodal perioperative temperature-management bundle was associated with a lower incidence of intraoperative hypothermia, shorter cumulative hypothermia duration, and less need for rescue rewarming in the PACU among patients undergoing relatively prolonged minimally invasive radical rectal cancer surgery. Composite complications were less frequent, and times to selected recovery milestones were shorter after implementation. Nonrandomized calendar-period allocation, bundled exposure, and potential secular confounding limit causal interpretation. Multicenter prospective studies are required to evaluate external applicability, necessary components, and cost-effectiveness.

## Data Availability

The original contributions presented in the study are included in the article/**Supplementary Material**. Further inquiries can be directed to the corresponding author.
