# Supplementary material for: Hypothermia and recovery outcomes before and after implementation of a multimodal perioperative temperature-management bundle in minimally invasive radical rectal cancer surgery
Source: Front Oncol. 2026 Aug 27;16:1888554. doi: 10.3389/fonc.2026.1888554 (PMC13558117; doi:10.3389/fonc.2026.1888554)
Supplement: Supplementary file 1 [file Table1.docx]

**Supplementary Materials**

**Supplementary Table 1. Temperature Data Quality, Bundle Adherence, Rescue Warming, and Postanesthesia Care Unit Transitions**

| **Module/metric** | **Pre-implementation cohort or overall cohort** | **Post-implementation cohort** | **Definition/handling** |
| --- | --- | --- | --- |
| Temperature data: included patients | 247 patients | — | Primary outcome ascertainable in all patients |
| AIMS sampling time points identified | 47,268 | — | From the first stable nasopharyngeal recording to probe removal |
| Valid temperature records | 46,937 (99.30%) | — | Passed prespecified quality-control rules |
| Missing or artifactual records | 331 (0.70%) | — | Physiologic-range, abrupt-change, and probe-disconnection rules |
| Patients with intermittent gaps | 41 patients (16.6%) | — | Longest consecutive gap, 3 min |
| Availability of T₁–T₄ windows | 247/247 (100%) | — | At least one valid value within ± 2 min of the target time |
| Residual diagnostics for the repeated-measures mixed model | No substantial deviation | — | Q–Q and residual-versus-fitted plots |
| Included cohort with > 20% key temperature data missing | 0 patients | — | Five additional patients were excluded at screening for this reason |
| Primary-outcome reclassification after interpolation | 0 patients | — | Interpolation was used only for cumulative duration |
| Intraoperative rescue warming before implementation | 81/84 (96.4%) | — | Initiated after temperature < 36.0°C for 15 min or immediately at < 35.5°C |
| Rescue warming not initiated | 3/84 (3.6%) | — | Hypothermia lasted < 15 min and minimum temperature was ≥ 35.5°C |
| Time from first temperature < 36.0°C to warming initiation (min) | 18 (12–27) | — | Among the 81 treated patients; includes the trigger-observation period |
| Time from fulfillment of the prespecified trigger to device activation (min) | 3 (2–5) | — | Among the 81 treated patients; reflects device-deployment delay |
| Duration of rescue warming (min) | 64 (39–96) | — | Among the 81 treated patients |
| Management of post-implementation hypothermia | — | Device and coverage checks completed in 19/19 | Scheduled active warming was continued |
| Duration of forced-air warming (min) | — | 197.3 ± 38.4 | Bundle adherence |
| Duration of circulating-water warming (min) | — | 215.8 ± 39.6 | Bundle adherence |
| Fluid-warmer output temperature (°C) | — | 37.0 ± 0.2 | Target, 37°C |
| CO₂-device output temperature (°C) | — | 37.1 ± 0.3 | Target, 37°C |
| CO₂-device output humidity (%) | — | 96.8 ± 2.1 | Target, > 95% |
| Fresh-gas flow (L/min) | 2.1 ± 0.6 | 0.7 ± 0.2 | Bundle component; *P* < 0.001 |
| Intraoperative hypothermia and hypothermia at PACU admission | 83 | 5 | Event persisted to PACU admission |
| Intraoperative hypothermia but normothermia at PACU admission | 1 | 14 | Rewarming before or during transfer |
| No intraoperative hypothermia but hypothermia at PACU admission | 13 | 0 | New onset during transfer |
| Normothermia intraoperatively and at PACU admission | 26 | 105 | No hypothermia |

**Note:** Data are presented as mean ± standard deviation (SD), median (interquartile range [IQR]), or number (percentage). AIMS, anesthesia information management system; PACU, postanesthesia care unit; CO₂, carbon dioxide; T₁–T₄, nasopharyngeal temperatures 30 min after anesthesia induction, 60 min after skin incision, 120 min after skin incision, and at the end of surgery; Q–Q, quantile–quantile. Temperature records and rescue interventions were reviewed according to prespecified rules. Intraoperative nasopharyngeal temperature and PACU tympanic temperature represent outcomes from different time windows.

**Supplementary Table 2. Conditional Subgroup Analysis Among Patients Who Developed Intraoperative Hypothermia**

| **Metric** | **Pre-implementation hypothermia subgroup (*n* = 84)** | **Post-implementation hypothermia subgroup (*n* = 19)** | **Statistical method** | **Raw *P*** | **Holm-adjusted *P*** |
| --- | --- | --- | --- | --- | --- |
| Duration of hypothermia (min) | 78.4 (42.5–118.6) | 24.3 (15.2–38.7) | Mann–Whitney *U* | < 0.001 | < 0.001 |
| Minimum intraoperative temperature (°C) | 34.92 ± 0.38 | 35.74 ± 0.29 | Welch *t* = −10.460 | < 0.001 | < 0.001 |
| Timing of minimum temperature ( ≤ 60 min/61–120 min/ > 120 min after incision to probe removal), *n* | 21/47/16 | 11/6/2 | Fisher–Freeman–Halton exact test | 0.023 | 0.023 |

**Note:** Data are presented as mean ± standard deviation (SD), median (interquartile range [IQR]), or number. *n*, number of patients. Hypothermia duration was compared using the Mann–Whitney *U* test; minimum temperature, using Welch’s *t* test; and timing of minimum temperature, using the Fisher–Freeman–Halton exact test. The three comparisons were adjusted using the Holm method. This conditional subgroup describes event severity; minimum temperature and cumulative duration in the full cohort were the primary measures of hypothermia burden.

**Supplementary Table 3. Full Distributions of Shivering Grades and Riker Sedation–Agitation Scale (SAS) Scores**

| **Metric** | **Pre-implementation cohort** | **Post-implementation cohort** | **Statistical method** | ***P* value** |
| --- | --- | --- | --- | --- |
| Shivering grade 0/1/2/3/4, *n* | 71/17/16/11/8 | 115/5/3/1/0 | Mann–Whitney *U*; Holm adjustment | < 0.001 |
| Riker SAS score 1/2/3/4/5/6/7, *n* | 0/2/9/72/25/12/3 | 0/2/18/94/8/2/0 | Mann–Whitney *U*; Holm adjustment | < 0.001 |

**Note:** Data are numbers at each grade. SAS, Riker Sedation–Agitation Scale; *n*, number of patients. Shivering was assessed on the Crossley–Mahajan ordered 0–4 scale, and SAS on the ordered 1–7 scale. Both complete ordinal distributions were compared using the Mann–Whitney *U* test with Holm adjustment. The main text separately reports the threshold outcomes of shivering (grade ≥ 1), agitation (SAS ≥ 5), and sedated state (SAS ≤ 3).

**Supplementary Table 4. Exploratory Hematologic, Coagulation, and Inflammatory Markers**

| **Marker** | **Time point** | **Pre-implementation cohort** | **Post-implementation cohort** | **Raw *P*** | ***q* value** |
| --- | --- | --- | --- | --- | --- |
| Hb (g/L) | Preoperative | 132.4 ± 18.6 | 131.8 ± 17.9 | 0.796 | — |
| Hb | 24 h | 104.2 ± 16.8 | 109.7 ± 15.3 | 0.008 | 0.009 |
| Hb | 72 h | 98.6 ± 15.4 | 104.8 ± 14.7 | 0.001 | 0.002 |
| PLT (×10⁹/L) | Preoperative | 218.6 ± 68.4 | 221.3 ± 72.1 | 0.763 | — |
| PLT | 24 h | 198.4 ± 62.3 | 204.7 ± 64.8 | 0.437 | 0.437 |
| PLT | 72 h | 242.8 ± 78.4 | 256.3 ± 82.1 | 0.188 | 0.200 |
| PT (s) | Preoperative | 12.1 ± 1.2 | 12.0 ± 1.1 | 0.496 | — |
| PT | 24 h | 15.2 ± 2.1 | 13.7 ± 1.8 | < 0.001 | < 0.001 |
| PT | 72 h | 13.4 ± 1.6 | 12.8 ± 1.4 | 0.002 | 0.003 |
| APTT (s) | Preoperative | 28.4 ± 3.8 | 28.1 ± 3.6 | 0.525 | — |
| APTT | 24 h | 37.8 ± 5.4 | 34.2 ± 4.6 | < 0.001 | < 0.001 |
| APTT | 72 h | 32.6 ± 4.2 | 30.4 ± 3.8 | < 0.001 | < 0.001 |
| FIB (g/L) | Preoperative | 3.24 ± 0.68 | 3.21 ± 0.65 | 0.723 | — |
| FIB | 24 h | 2.48 ± 0.62 | 2.78 ± 0.58 | < 0.001 | < 0.001 |
| FIB | 72 h | 3.12 ± 0.72 | 3.38 ± 0.68 | 0.004 | 0.005 |
| CRP (mg/L) | Preoperative | 4.8 (2.3–8.6) | 4.6 (2.1–8.2) | 0.812 | — |
| CRP | 24 h | 72.4 (48.6–98.3) | 54.8 (36.2–78.4) | < 0.001 | < 0.001 |
| CRP | 72 h | 42.8 (28.6–62.4) | 28.4 (18.3–44.7) | < 0.001 | < 0.001 |
| IL-6 (pg/mL) | Preoperative | 9.8 (6.4–15.2) | 9.6 (6.2–14.8) | 0.847 | — |
| IL-6 | 24 h | 88.6 (62.4–124.8) | 64.3 (45.2–96.7) | < 0.001 | < 0.001 |
| IL-6 | 72 h | 38.4 (24.6–58.7) | 26.8 (16.4–42.3) | < 0.001 | < 0.001 |
| PCT (ng/mL) | Preoperative | 0.05 (0.03–0.08) | 0.05 (0.03–0.07) | 0.924 | — |
| PCT | 24 h | 0.22 (0.12–0.38) | 0.14 (0.08–0.24) | < 0.001 | < 0.001 |
| PCT | 72 h | 0.09 (0.05–0.14) | 0.06 (0.04–0.10) | 0.003 | 0.004 |

**Note:** Data are presented as mean ± standard deviation (SD) or median (interquartile range [IQR]). Hb, hemoglobin; PLT, platelet count; PT, prothrombin time; APTT, activated partial thromboplastin time; FIB, fibrinogen; CRP, C-reactive protein; IL-6, interleukin-6; PCT, procalcitonin; FDR, false discovery rate; *q*, FDR-adjusted *P* value. Normally distributed variables were compared using the independent-samples *t* test and skewed variables using the Mann–Whitney *U* test. The 16 postoperative comparisons were adjusted using the Benjamini–Hochberg FDR procedure; preoperative values were used only to describe baseline and were not included in the postoperative comparison family.

**Supplementary Table 5. Supplementary Volume, Blood Loss, and Red Blood Cell Transfusion Metrics**

| **Metric** | **Pre-implementation cohort** | **Post-implementation cohort** | **Test statistic** | ***P* value** |
| --- | --- | --- | --- | --- |
| Intraoperative blood loss (mL) | 142.3 ± 68.5 | 138.7 ± 65.2 | *t* = 0.423 | 0.673 |
| Intraoperative red blood cell transfusion, n (%) | 6 (4.9) | 5 (4.0) | Fisher's exact test | 0.769 |
| Red blood cell transfusion volume (mL; transfused patients only) | 300 (300–450) | 300 (300–450) | Descriptive | — |
| Postoperative intravenous fluid volume, 0–24 h (mL) | 1885 ± 405 | 1848 ± 398 | *t* = 0.724 | 0.470 |
| Postoperative urine output, 0–24 h (mL) | 1268 ± 392 | 1296 ± 401 | *t* = −0.555 | 0.579 |
| Postoperative intra-abdominal drainage, 0–24 h (mL) | 162 ± 68 | 158 ± 64 | *t* = 0.476 | 0.635 |
| Recorded fluid balance, 0–24 h (intravenous input − urine output − intra-abdominal drainage, mL) | +455 ± 315 | +394 ± 301 | *t* = 1.556 | 0.121 |
| Additional red blood cell transfusion, postoperative 0–72 h, n (%) | 5 (4.1) | 4 (3.2) | Fisher's exact test | 0.749 |

**Note:** Data are presented as mean ± standard deviation (SD), median (interquartile range [IQR]), or number (percentage). *n*, number of patients; h, hours. Continuous variables were compared using the independent-samples *t* test, and binary transfusion outcomes using Fisher’s exact test. Recorded 0–24 h fluid balance equals intravenous input minus urine output minus intra-abdominal drainage and does not represent complete volume status. Because few patients received an intraoperative red blood cell transfusion, transfusion volume is reported descriptively.

**Supplementary Table 6. Multiplicity-Control Inventory**

| **Outcome family** | **Outcome** | **Raw *P*** | **Adjusted *P*/*q*** | **Interpretation** |
| --- | --- | --- | --- | --- |
| Primary outcome | Intraoperative hypothermia | < 0.001 | No adjustment | Significant |
| Key secondary outcome | Minimum intraoperative temperature (full cohort) | < 0.001 | Holm < 0.001 | Significant |
| Key secondary outcome | Cumulative time with temperature < 36.0°C | < 0.001 | Holm < 0.001 | Significant |
| Key secondary outcome | PACU length of stay | < 0.001 | Holm < 0.001 | Significant |
| Key secondary outcome | Shivering | < 0.001 | Holm < 0.001 | Significant |
| Key secondary outcome | Composite complications | < 0.001 | Holm 0.001 | Significant |
| Intraoperative time point | T₁ between-group comparison | < 0.001 | Holm < 0.001 | Significant |
| Intraoperative time point | T₂ between-group comparison | < 0.001 | Holm < 0.001 | Significant |
| Intraoperative time point | T₃ between-group comparison | < 0.001 | Holm < 0.001 | Significant |
| Intraoperative time point | T₄ between-group comparison | < 0.001 | Holm < 0.001 | Significant |
| Other PACU outcome | T₅ admission temperature | < 0.001 | BH *q* < 0.001 | Significant |
| Other PACU outcome | Hypothermia at PACU admission | < 0.001 | BH *q* < 0.001 | Significant |
| Other PACU outcome | T₆ discharge temperature | < 0.001 | BH *q* < 0.001 | Significant |
| Other PACU outcome | Agitation | < 0.001 | BH *q* < 0.001 | Significant |
| Other PACU outcome | Sedated state | 0.088 | BH *q* = 0.096 | Not significant |
| Other PACU outcome | Rescue analgesia | < 0.001 | BH *q* < 0.001 | Significant |
| Other PACU outcome | Postoperative nausea and vomiting | 0.096 | BH *q* = 0.096 | Not significant |
| Individual complication | Incisional surgical-site infection | 0.030 | BH *q* = 0.181 | Not significant |
| Individual complication | Anastomotic leakage | 0.568 | BH *q* = 0.568 | Not significant |
| Individual complication | Pulmonary infection | 0.076 | BH *q* = 0.227 | Not significant |
| Individual complication | Urinary tract infection | 0.384 | BH *q* = 0.568 | Not significant |
| Individual complication | Deep vein thrombosis | 0.500 | BH *q* = 0.568 | Not significant |
| Individual complication | Cardiovascular event | 0.446 | BH *q* = 0.568 | Not significant |
| Recovery | Time to first flatus | < 0.001 | BH *q* < 0.001 | Significant |
| Recovery | Time to first oral intake | < 0.001 | BH *q* < 0.001 | Significant |
| Recovery | Time to first ambulation | < 0.001 | BH *q* < 0.001 | Significant |
| Recovery | Urinary catheter duration | < 0.001 | BH *q* < 0.001 | Significant |
| Recovery | Time to drain removal | < 0.001 | BH *q* < 0.001 | Significant |
| Recovery | Postoperative length of stay | < 0.001 | BH *q* < 0.001 | Significant |
| Pain | Resting NRS at 24 h | < 0.001 | BH *q* < 0.001 | Significant |
| Pain | Resting NRS at 48 h | < 0.001 | BH *q* < 0.001 | Significant |
| Pain | Total opioid consumption | < 0.001 | BH *q* < 0.001 | Significant |
| Laboratory | Hb at 24 h | 0.008 | BH *q* = 0.009 | Exploratory; significant |
| Laboratory | Hb at 72 h | 0.001 | BH *q* = 0.002 | Exploratory; significant |
| Laboratory | PLT at 24 h | 0.437 | BH *q* = 0.437 | Not significant |
| Laboratory | PLT at 72 h | 0.188 | BH *q* = 0.200 | Not significant |
| Laboratory | PT at 24 h | < 0.001 | BH *q* < 0.001 | Exploratory; significant |
| Laboratory | PT at 72 h | 0.002 | BH *q* = 0.003 | Exploratory; significant |
| Laboratory | APTT at 24 h | < 0.001 | BH *q* < 0.001 | Exploratory; significant |
| Laboratory | APTT at 72 h | < 0.001 | BH *q* < 0.001 | Exploratory; significant |
| Laboratory | FIB at 24 h | < 0.001 | BH *q* < 0.001 | Exploratory; significant |
| Laboratory | FIB at 72 h | 0.004 | BH *q* = 0.005 | Exploratory; significant |
| Laboratory | CRP at 24 h | < 0.001 | BH *q* < 0.001 | Exploratory; significant |
| Laboratory | CRP at 72 h | < 0.001 | BH *q* < 0.001 | Exploratory; significant |
| Laboratory | IL-6 at 24 h | < 0.001 | BH *q* < 0.001 | Exploratory; significant |
| Laboratory | IL-6 at 72 h | < 0.001 | BH *q* < 0.001 | Exploratory; significant |
| Laboratory | PCT at 24 h | < 0.001 | BH *q* < 0.001 | Exploratory; significant |
| Laboratory | PCT at 72 h | 0.003 | BH *q* = 0.004 | Exploratory; significant |
| Conditional hypothermia subgroup | Duration of hypothermia | < 0.001 | Holm < 0.001 | Exploratory; significant |
| Conditional hypothermia subgroup | Minimum intraoperative temperature | < 0.001 | Holm < 0.001 | Exploratory; significant |
| Conditional hypothermia subgroup | Timing of minimum temperature | 0.023 | Holm 0.023 | Exploratory; significant |
| Supplementary ordinal scale | Distribution of shivering grades 0–4 | < 0.001 | Holm < 0.001 | Exploratory; significant |
| Supplementary ordinal scale | Distribution of SAS scores 1–7 | < 0.001 | Holm < 0.001 | Exploratory; significant |

**Note:** The Holm method controlled the family-wise error rate for the key secondary outcomes and other specified families. The Benjamini–Hochberg (BH) false discovery rate (FDR) procedure was used for other clinically defined outcome families; *q* denotes the FDR-adjusted *P* value. The primary outcome was not adjusted. All adjustments used unrounded raw *P* values, and two-sided *P* or *q* < 0.05 was considered statistically significant. PACU, postanesthesia care unit; T₁–T₆, prespecified temperature time points; NRS, Numeric Rating Scale; Hb, hemoglobin; PLT, platelet count; PT, prothrombin time; APTT, activated partial thromboplastin time; FIB, fibrinogen; CRP, C-reactive protein; IL-6, interleukin-6; PCT, procalcitonin; SAS, Riker Sedation–Agitation Scale.

**Supplementary Table 7. Regression Model Diagnostics and Sensitivity Analyses**

| **Diagnostic/sensitivity analysis** | **Result** | **95% CI or range** | ***P* value/interpretation** |
| --- | --- | --- | --- |
| Number of events/number of predictor parameters | 103/8; events per predictor parameter = 12.9 | — | Stability assessed using penalized regression and bootstrap |
| Maximum VIF | 1.84 | 1.03–1.84 | No substantial collinearity |
| Linearity of continuous variables in the logit | No significant deviation | All tests *P* > 0.10 | Model assumption acceptable |
| Forced-entry logistic regression: implementation-period OR | 0.066 | 0.029–0.149 | *P* < 0.001 |
| Firth-penalized logistic regression: implementation-period OR | 0.071 | 0.032–0.157 | *P* < 0.001 |
| 1000 bootstrap resamples: median implementation-period OR | 0.068 | Percentile 95% interval, 0.031–0.151 | Interval did not include 1 |
| Baseline-only sensitivity model: implementation-period OR | 0.061 | 0.027–0.139 | *P* < 0.001 |

**Note:** OR, odds ratio; CI, confidence interval; VIF, variance inflation factor; logit, log-odds function in binary logistic regression; Firth regression, penalized-likelihood binary logistic regression; bootstrap, repeated resampling with replacement. Firth regression and 1000 bootstrap resamples used the extended clinical association covariate set; the baseline-only sensitivity model included only variables available before implementation-period assignment. The events-per-predictor-parameter ratio was calculated from 103 hypothermia events and eight predictor parameters.
